# Elucidation of DNA-Eltrombopag Binding: Electrochemical, Spectroscopic and Molecular Docking Techniques

**DOI:** 10.3390/bios13030300

**Published:** 2023-02-21

**Authors:** Somaye Cheraghi, Pelin Şenel, Burcu Dogan Topal, Soykan Agar, Mahsa Majidian, Mine Yurtsever, Esen Bellur Atici, Ayşegül Gölcü, Sibel A. Ozkan

**Affiliations:** 1 Department of Analytical Chemistry, Faculty of Pharmacy, Ankara University, Ankara 06560, Turkey; 2 Department of Chemistry, Shahid Bahonar University of Kerman, Kerman 7616914111, Iran; 3 Department of Chemistry, Faculty of Science and Letters, Istanbul Technical University, Istanbul 34469, Turkey; 4 R&D Center, DEVA Holding A.S., Tekirdağ 59510, Turkey

**Keywords:** eltrombopag, biosensor, DNA, voltammetry, molecular docking

## Abstract

Eltrombopag is a powerful adjuvant anticancer drug used in treating MS (myelodysplastic syndrome) and AML (acute myeloid leukemia) diseases. In this study, the interaction mechanism between eltrombopag and DNA was studied by voltammetry, spectroscopic techniques, and viscosity measurements. We developed a DNA-based biosensor and nano-biosensor using reduced graphene oxide-modified glassy carbon electrode to detect DNA-eltrombopag binding. The reduction of desoxyguanosine (dGuo) and desoxyadenosine (dAdo) oxidation signals in the presence of the drug demonstrated that a strong interaction could be established between the eltrombopag and dsDNA. The eltrombopag-DNA interaction was further investigated by UV absorption and fluorescence emission spectroscopy to gain more quantitative insight on binding. Viscosity measurements were utilized to characterize the binding mode of the drug. To shed light on the noncovalent interactions and binding mechanism of eltrombopag molecular docking and molecular dynamics (MD), simulations were performed. Through simultaneously carried out experimental and in silico studies, it was established that the eltrombopag binds onto the DNA via intercalation.

## 1. Introduction

Eltrombopag (ELT), C_25_H_22_N_4_O_4_, is a thrombopoietin-receptor agonist, frequently used in the treatment of chemotherapy-induced chronic immune (idiopathic) thrombocytopenic purpura (ITP) and aplastic anemia [1,2]. Determining its concentration in the blood of the patients is necessary due to undesired side effects such as thromboembolism risks and liver dysfunction as a result of overdose [2]. In addition, ELT persuades platelet production and reduces bleeding complications by stimulating the thrombopoietin receptor.

The interaction of DNA with drugs is significant in pharmaceutical development processes. DNA-drug binding may occur in many ways, such as intercalation, non-covalent binding, covalent binding, nucleoside-analog incorporation, and DNA cleaving [3]. The interaction between drugs and DNA can be detected using voltammetry [4,5], spectroscopy [6,7], high-performance liquid chromatography (HPLC) [8], surface plasmon resonance [9], and molecular modeling methods [10]. In addition, DNA-based electrochemical biosensors have been recently selected due to their excellent attributes, such as easy fabrication, high sensitivity, selectivity, and rapidness [11,12,13,14]. The development of a nanobiosensor, which is a miniaturized device, has been a requirement for studying DNA-drug interactions.

Nanomaterials, particularly carbon-based nanomaterials such as carbon nanotubes and graphene, are a great choice for improving the performance of DNA biosensors for the determination of anticancer drugs [15,16,17]. These materials make the electrode surface more sensitive because they contain the active sites in their structure, which increases the electron transfer rate and the possibility of reacting at lower potentials. Graphene oxide (GO) and reduced graphene oxide (rGO) are suitable materials for application in supercapacitors, electronics, nanocomposites, batteries, and sensors owing to their high surface area, high mechanical properties, fast electron transfer, good electric conductivity, and low cost [18]. Because of these advantages, it can be used to modify the sensors for electrochemical methods. We designed a nanobiosensor based on synthesized rGO for the detection of DNA in low concentrations and investigation of DNA-drug interactions. There are no electrochemical studies on the DNA-ELT interaction in the literature.

This work performed the interaction between dsDNA and ELT on a dsDNA biosensor and dsDNA nanobiosensor. All experimental parameters such as concentration of nanomaterial suspension, dropping volume, interaction time, and concentration were optimized. The evaluation of the interaction mechanism between dsDNA and ELT was also investigated by comparative experimental and theoretical studies.

## 2. Materials and Methods

### 2.1. Electrochemical Studies

#### 2.1.1. Materials and Apparatus

Calf thymus (ct)-dsDNA were purchased from Sigma-Aldrich. A standard stock solution of ELT was prepared by dissolving 0.005 g ELT (purchased from DEVA Holding- Tekirdag-Turkiye) in 10 mL ethanol. Acetic acid and sodium acetate were obtained from Merck and were used to prepare the acetate buffer solution. GO was synthesized according to the Hummer method and a previously published article [19,20]; it was converted to rGO by the thermal reduction method [21], and using chemicals including graphite powder, sulfuric acid, hydrochloric acid, potassium permanganate, sodium nitrate, and hydrogen peroxide purchased from Merck Company. The AUTOLAB electrochemical apparatus was used in conjunction with the NOVA 2.1.5 software analyzer for all the voltammetric studies. We used Ag/AgCl as a reference electrode, Pt wire as an auxiliary electrode, and glassy carbon electrode (GCE) as a working electrode. Differential pulse voltammograms (DPV) were recorded under the following conditions: step potential: 0.002 V; modulation amplitude: 0.05 V; modulation time: 0.07 s; interval time: 0.4 s.

#### 2.1.2. Fabrication of DNA/rGO/GCE 

**Scheme 1:**
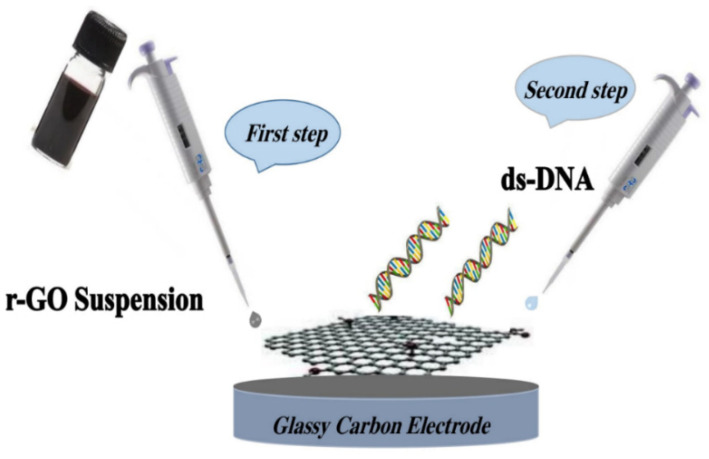
The fabrication procedure of DNA/rGO/GCE.

Firstly, GO was synthesized according to the Hummer method and a previously published article [19,20]; it was then converted to rGO by the thermal reduction method [21]. We mixed 0.02 g of GO powder in 50 mL of deionized water for half an hour in an ultrasonic bath. Then, the suspension mixture was filtered and dried at 80 °C for 24 h to produce rGO. At this stage, GO was transformed into reduced graphene by removing the hydrogen from the hydroxyl groups. To prepare DNA/rGO/GCE, GCE was first polished via Al_2_O_3_ powder for 5 min and rinsed with distilled water. The electrode was made by layer-by-layer modification. A suspension of rGO nanoparticles was prepared by adding 1 mg of nanomaterials to a water/ethanol (1:1) solution. This suspension was then transferred to an ultrasonic bath for 1 h; a heterogenous nanoparticles suspension was obtained. Next, various amounts of rGO suspension were dropped onto the GCE surface in the range of 1–10 µL. The optimum amount of 2 µL suspension was dropped on the surface of GCE and dried at 40 °C. Then, a layer of ds-DNA was placed on the electrode surface by dropping 5 µL of ds-DNA solution (50 mg/L), and was left to dry at 35 °C. The DNA/rGO/GCE fabrication process is depicted in Scheme 1.

### 2.2. Spectrophotometric Studies

All chemicals (CH_3_COOH, NaOH, Tris-HCl, NaCl, dsDNA (fish sperm)), Hoechst 33258, and EtBr used in the spectroscopic studies were purchased from Sigma-Aldrich. The molar concentration of DNA used in the binding studies with dsDNA was studied similarly to the standard procedure we used in our previous studies [21]. However, in this study, 383 µM dsDNA stock solution was used and the A_260_/A_280_ value was calculated as 1.93, and the epsilon (ε) value was taken as 6600 M^−1^cm^−1^ in all calculations [21]. The stock solutions of Hoechst-33258 and ethidium bromide (EtBr) were prepared in buffer solution (including 0.2 M Tris-HCl and 150 mM NaCl at pH 7.4). We prepared 1 × 10^−3^ M ELT stock solution in ethanol. The solution was used after keeping it in the refrigerator for 16 h. The stock was used for a maximum of two days.

### 2.3. Physical Measurements and Instrumentation

All information about physical measurements and instrumentation are summarized in Table 1. The experiments of all techniques are the same as those we used in our previous studies [21], with only the concentrations being different. All these differences are summarized in Table 1.

The viscosity tests were performed under the same experimental procedures and equations used in the previous study [21]. All terms given in the equations have the same meaning [22].

All test results at 25 °C were obtained in three replicates. The *K*_b_ values obtained for all methods are given by relative standard deviation (RSD).

### 2.4. Molecular Docking Simulations

#### 2.4.1. DNA Receptor Preparation 

For docking simulations, a typical human DNA (B-DNA) was represented by a dodecamer chain (5′D(CGCGAATTCGCG)-3′) whose crystal structure was retrieved from the protein data bank with **PDB ID: 1BNA**. Crystal water molecules were cleaned, and the missing hydrogen atoms were completed. A grid box of 58, 36, 86 points along x, y, z directions, respectively, was built around the center of the DNA structure. The prepared DNA segment (1BNA dodecamer) was placed into the grid box.

#### 2.4.2. ELT Ligand Preparation

XRD structure of ELT (ChEMBL ID: 461101) was retrieved from the ChEMBL database. Missing hydrogen atoms were completed. The protonation state of ELT was adjusted to the physiological pH of 7.4. Avogadro software was utilized together with Gasteiger–Marsili charges. Autodock Vina 1.1.2 software was used for blind molecular docking studies which has been repeated 10 times. We performed 100 posed cluster simulations (1000 poses in total) and the docking scores were tabulated as the free energy of binding. The energetically most favorable structures were then analyzed in terms of binding region of the drug and the interactions it forms with DNA nucleotides.

### 2.5. Molecular Dynamics (MD) Simulations

MD simulations were carried out using Desmond software. The stability and dynamic properties of the drug-DNA complex were monitored. The dimensions of the orthorhombic simulation box were chosen to be 30 × 30 × 30 Å^3^. The TIP3P force field for water was employed. To neutralize the medium, 0.15 M Na^+^ and Cl^−^ ions were added into the box. The system with approximately 77,000 atoms were subjected to MD simulations at the NPT ensemble at 310 K, 1.01 bar. The initial structure of the atoms was taken from the docking poses with the lowest energy. The Nose-Hoover thermostat and Martyna Tobias−Klein barostat were used to keep temperature and pressure constant. The systems were simulated for 50 ns, which was enough to reach equilibrium. The hydrogen bond contact analyses were done using the stored trajectory data. The RMSD graphs for 1BNA with and without ELT were plotted.

## 3. Results & Discussion

### 3.1. Electrochemical Studies

#### 3.1.1. Interaction Studies at the Biosensor

**Figure 1:**
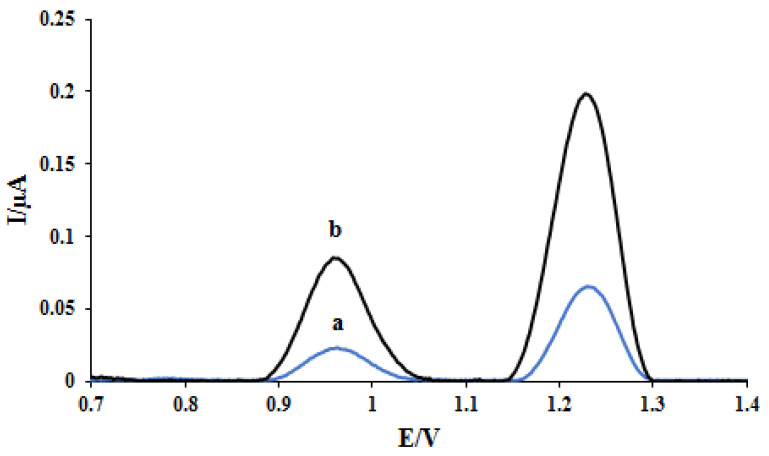
DPVs of (a) DNA/GCE and (b) DNA/rGO/GCE in pH 4.7 acetate buffer.

In the studies of DNA-based electrochemical sensors, the reduction of dAdo and dGuo oxidation peak current and the potential shifting of these bases signals verify the interaction of the drug and the DNA double helix bonds [16]. As seen in Figure 1, at the biosensor, the oxidation peak potential of desoxyguanosine (dGuo) and desoxyadenosine (dAdo) was obtained at 0.96 V and 1.23 V, respectively. The effect of interaction time and concentration of ELT on the voltammetric peak of dGuo and dAdo were investigated by differential pulse voltammetry. The optimization procedure of interaction time was utilized in the range of 15–600 s in the presence of constant 12.0 ppm ELT, and 300 s was found as the optimum value (not shown). After selecting the interaction time, the effect of concentration on the voltammetric peak dGuo and dAdo was evaluated in the range of 1.0–8.0 ppm. The decrease of peak currents of dGuo and dAdo was observed until 2 ppm. After 2 ppm, there was no change in the voltammetric signals of the dsDNA bases.

#### 3.1.2. Interaction Studies at Nanobiosensor

##### Characterization of DNA/rGO/GCE

In the previously published article [20], the atomic force microscope (AFM) images of a bare GCE and GO-modified GCE were reported.

Figure 1 shows the effect of modification of the electrode surface with rGO nanoparticles on the performance of the electrode using the DPV method in acetate buffer solution (0.5 M, pH 4.8). Increasing the active surface of the electrode after modification with rGO nanoparticles causes better adsorption of DNA at the electrode surface. Furthermore, the high electrical conductivity of rGO nanoparticles increased the oxidation peak current of dGuo and dAdo at the nano-biosensor surface (curve b) compared with the bare electrode (curve a).

##### Electrochemical Investigation of ELT Interaction with DNA

**Figure 2:**
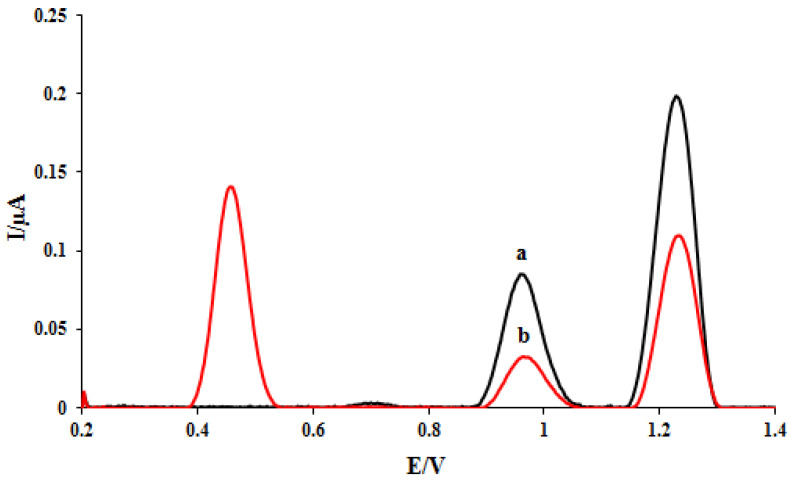
DPVs of DNA/rGO/GCE in the absence (a) and the presence of 3 mg L^−1^ ELT (b).

The RSD values (*n* = 3) of 4.43 and 4.6 were obtained for the dGuo and dAdo oxidation currents, which confirms the repeatability of DNA/rGO/GCE. The interaction between ELT and DNA was investigated at the DNA/rGO/GCE surface. To evaluate the ELT interaction with DNA, the DPVs of the proposed nanobiosensor were recorded in the absence and presence of 3 mg/L of the ELT (Figure 2). The electrochemical oxidation of dGuo and dAdo bases at the DNA/rGO/GCE surface in acetate buffer solution (0.5 M, pH 4.8) yielded currents of 0.083 μA (potential. 0.96 V) and 0.19 μA (potential. 1.2 V), respectively (curve a). To investigate the ELT-DNA interaction, the electrode was immersed in a solution containing 3 mg/L of the drug for 5 min. The electrode was then washed with acetate buffer to remove unreacted ELT from the electrode surface and transferred to a cell containing clean acetate buffer (0.5 M, pH 4.8), and DP voltammograms were recorded. The response of ELT appeared at a potential of 0.45 V. The oxidation signals of dAdo and dGuo reduced to 0.032 μA and 0.1 μA, respectively, after interaction with the ELT (curve b).

**Figure 3:**
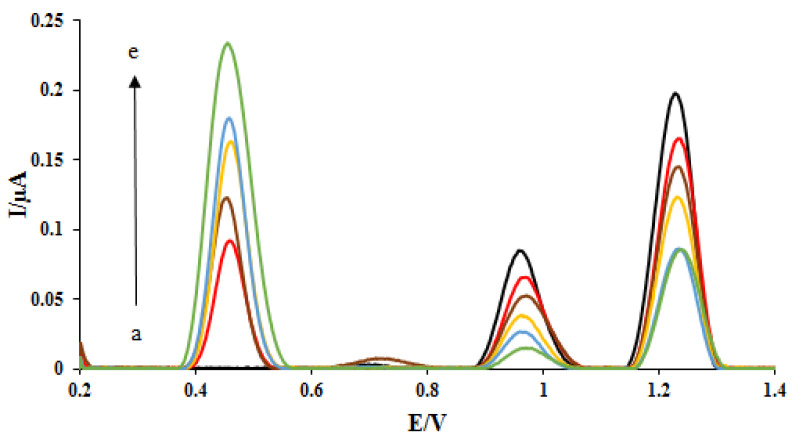
DPVs of DNA/rGO/GCE in acetate buffer solution (0.5 M, pH 4.8) containing 2 mg L^−1^ ELT under different incubation time: 15 s (a), 30 s (b), 1 min (c), 3 min (d), and 5 min (e). The arrow shows the increasing peak current of ELT with the interaction time.

**Figure 4:**
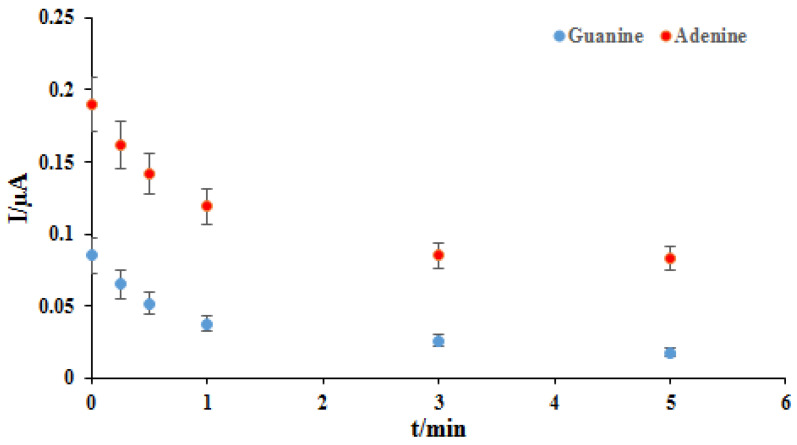
The plot of the guanine (blue dots) and adenine (red dots) peaks current vs. different interaction times with the ELT.

One of the essential and crucial parameters is the interaction time studies for drug-DNA binding. Therefore, using differential pulse voltammetry in the presence of 2 mg/L of the ELT, various interaction times were investigated in the range of 15–600 s (Figure 3). As seen in Figure 3, the oxidation peak currents of dGuo and dAdo were decreased with the increase of interaction times. The shifting of the peak potential of dGuo in a positive direction shows that the drug may be interacting with dsDNA via the intercalation mode. The plot of peak currents of dGuo (blue dots) and dAdo (red dots) versus interaction times was shown in Figure 4. At 5.0 min, selected as the optimum interaction time, the signal of dGuo and dAdo showed the minimum standard error.

**Figure 5:**
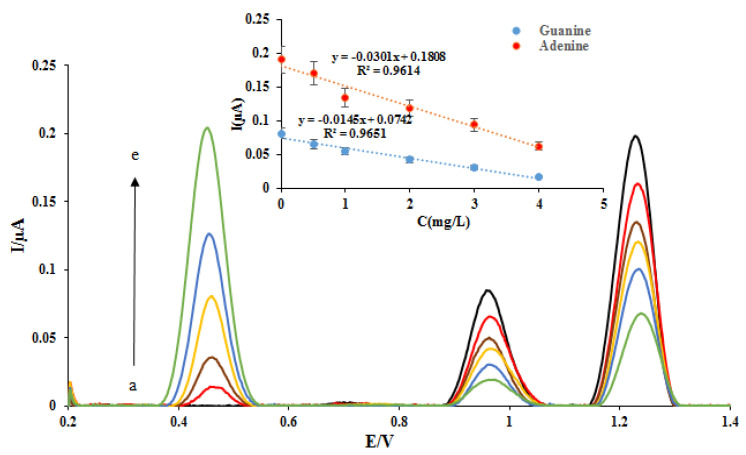
DPVs of DNA/rGO/GCE in the absence (black) and in the presence of (a) 0.5, (b) 1, (c) 2, (d) 3, and (e) 4 mg L^—1^ ELT. Insert: The plot of the peak currents of guanine (blue dots) and adenine (red dots) vs. ELT concentration.

Then, the measurement of different concentrations of the ELT at the proposed nano-biosensors surface was investigated in the range of 0.5–4.0 ppm. The DPVs of DNA/rGO/GCE were recorded in the absence and presence of different concentrations of the ELT (Figure 5). As can be seen, with increasing drug concentration, the signal related to the ELT oxidation (potential. 0.45 V) has increased. Because of the increase of the amount of drug onto the DNA double helix bonds and the ELT-DNA interaction increasing, the oxidation peak currents of dGuo and dAdo were decreased. The plots of the peak currents of dGuo (blue dots) and dAdo (red dots) versus ELT concentration are shown in Figure 5. The regression equation and determination coefficients (*R*^2^) for these plots were calculated and shown in the Figure 5 inset. Thanks to the rGO materials, the sensing performance of the nanobiosensor was enhanced compared with the biosensor. It is concluded that a lower concentration of ELT can be examined on a nanobiosensor based on the dsDNA-ELT binding.

### 3.2. Spectroscopic Studies

In order to fully understand the interaction mechanism of dsDNA with drug molecules, viscosimetric and spectroscopic techniques are frequently used in addition to voltammetry. For this purpose, UV-Vis and fluorescent spectroscopy experiments were performed in this study. In addition, thermal denaturation and viscosimetric analyses were also performed, and the results of all methods were interpreted to determine the DNA binding mode of the drug. ELT-dsDNA binding studies were first initiated with the UV-Vis absorption technique. This method recorded changes in the absorption spectrum of the ELT solution with increasing dsDNA additions. The interaction mechanism of ELT with a biomolecule such as DNA can actually change the absorption intensity and wavelengths depending on the severity and probability of this interaction. If there is an intervening binding state, a hypochromic or hyperchromic effect in the absorption intensities of the drug as well as changes in peak positions via red shift are expected. On the other hand, in the case of groove binding mode, the following are expected: (a) hypochromic effect in the absorption intensities of the drug and insignificant or no changes in the peak positions, and (b) hyperchromic effect in the absorption intensities of the drug and also changes in the peak positions through blue shift. Significant changes in ELT UV-vis bands certainly confirmed the dsDNA-ELT interaction. ELT/dsDNA mixed solutions were prepared by adding different dsDNA concentrations (20 to 140 µM) to 80 µM ELT solution.

**Figure 6:**
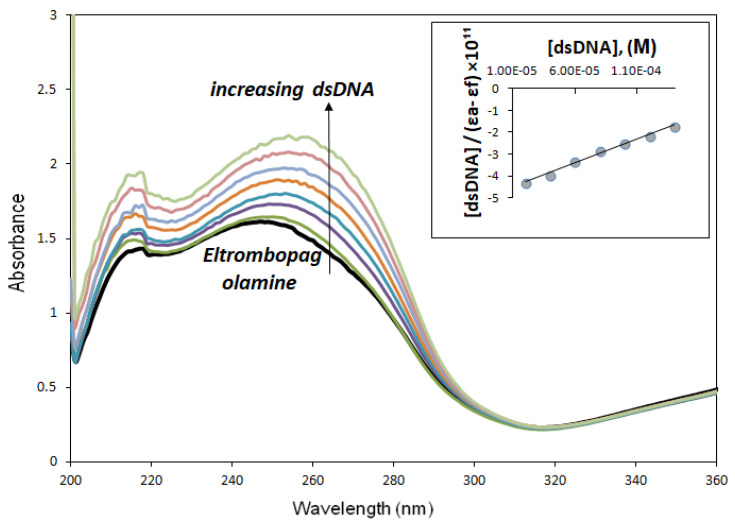
UV-Vis spectra of ELT (8 × 10^−6^M) upon the titration of dsDNA (20–140 × 10^−6^M) in buffer media. The increase in absorbance for an increase in dsDNA concentration is indicated by an “arrow”. The maximum wavelength of ELT (λ_max_ = 249 nm) shifted to 256 nm. *K_b_* was calculated from this linearity, showing the linear agreement between “[dsDNA]/(*ε_a_ − ε_f_*)” and [dsDNA] in the Appendix (*R*^2^ = 0.9930).

Upon adding different concentrations of dsDNA solutions to 80 µM ELT solution, the solution showed an increase in the intensities of the absorption peaks, indicating the expected hyperchromic effect of approximately 34.51 ± 0.29%, while the wavelengths of the maximum absorption peaks shifted by 7 nm called red shift. This is probably due to π → π* or n → π* transition. So here, the ELT molecule acting as a planar aromatic moiety was inserted non-covalently between adjacent DNA base pairs and caused DNA-ELT intercalation [23]. Figure 6 shows the absorption spectra of not only ELT but also its interaction with dsDNA. The binding constant (*K_b_*) of ELT, which indicates the strength of interaction with dsDNA, was determined using the equation below [24]:

$$\frac{\left[\mathrm{dsDNA}\right]}{\varepsilon_{a}-\varepsilon_{f}}=\frac{\left[\mathrm{dsDNA}\right]}{\varepsilon_{b}-\varepsilon_{f}}+\frac{1}{k_{b}\left(\varepsilon_{b}-\varepsilon_{f}\right)}$$

where ε*_a_* is the apparent extinction coefficient calculated using A_observed_/[ELT], and ε*_f_* is the molar extinction coefficient of the drug-free form that has not interacted with dsDNA. ε*_b_* is the molar extinction coefficient of the completely interacted drug with dsDNA, and [dsDNA] is the concentration of dsDNA in terms of base pairs. *K_b_* is the binding constant showing the binding of the ELT to dsDNA and was calculated from the slope of the line drawn between [dsDNA]/(ε*_a_* − ε*_f_*) and [dsDNA]. The *K_b_* value was 4.63 × 10^5^ ± 0.34 M^−1^ for ELT (log*K_b_* = 5.67 ± 0.13 M^−1^).

**Figure 7:**
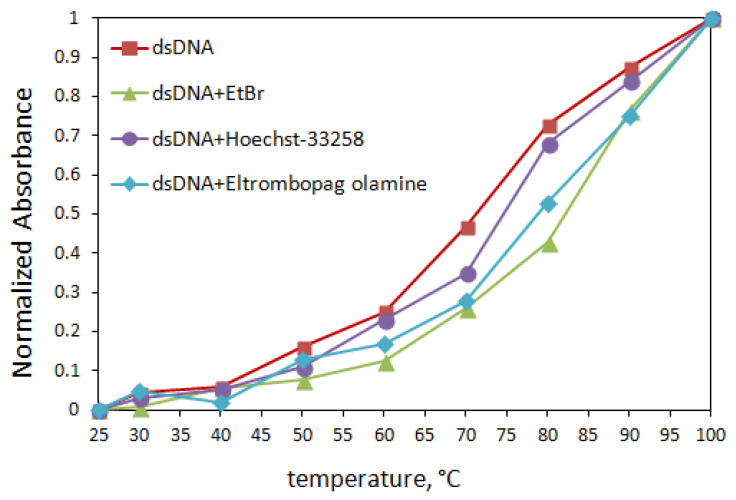
Thermal denaturation curves of dsDNA (120 µM) in the absence and in the presence of ELT and some intercalator/groove agents (10 µM) in Tris/HCl buffer solution.

In light of these data, the UV-Vis absorption results suggested that the binding mode was intercalation, but we needed to experiment with more methods to confirm. Therefore, thermal denaturation studies were performed with the same device. Two critical parameters must be calculated in denaturation studies used in the interaction of dsDNA with small molecules such as drugs. One of them is the melting temperature of dsDNA only (*T*^0^_m_) and the other one is *T*_m_ (indicates the melting temperature of ELT + dsDNA solutions). Denaturation studies only have one purpose. That is, when we gradually increase the temperature of the solution containing the double-stranded DNA we use in our studies, we observe the breaking of the hydrogen bonds between the base pairs of the double-stranded DNA and, at the same time, record the temperature at which dsDNA gradually begins to decompose into single strands. The thermal denaturation profiles of dsDNA in the absence and presence of ELT and some intercalator/groove agents are given in Figure 7.

It is known [25] that EtBr binds to dsDNA via intercalation because the stability of the complex formed in the EtBr + dsDNA mixed solution is higher than the groove binders (Rhodamin B: small groove binder, Hoechst-33258: small groove binder, and Methyl Green: large groove binder). This binding significantly increases the thermal denaturation temperature of dsDNA. In contrast, groove-binding agents cannot raise the denaturation temperature of dsDNA much, which we have confirmed in several studies [26,27,28,29]. Table 2 shows *T*_m_ values based on the interaction of dsDNA with different agents.

The results obtained from the thermal denaturation studies, as seen in Table 2, showed that the *T*_m_ value of the EtBr compound + dsDNA solution was 83.4 ± 0.08 °C, with an increase of 11.6 degrees Celsius. On the other hand, the Δ*T*_m_ value observed in a groove-binding agent such as Hoechst-33258 is almost the same, with an average increase of 5.6 degrees Celsius. In the absence and presence of ELT, the *T*_m_ value of 120 µM dsDNA was measured to be 72.2 ± 0.07 °C and 81.5 ± 0.18 °C, respectively. The 9.3 °C increase in temperature indicates that the addition of ELT caused an increase in solidity in the nucleic acid double helix like EtBr, so these results indicate that ELT interacts with dsDNA by intercalation. The 9.3 °C increase in temperature with the addition of the solution containing ELT to dsDNA showed that this drug active ingredient also caused an increase in stability in the double helix structure of deoxyribonucleic acid like EtBr. Based on these data, we can conclude that ELT can bind to dsDNA by the intercalation mode.

**Figure 8:**
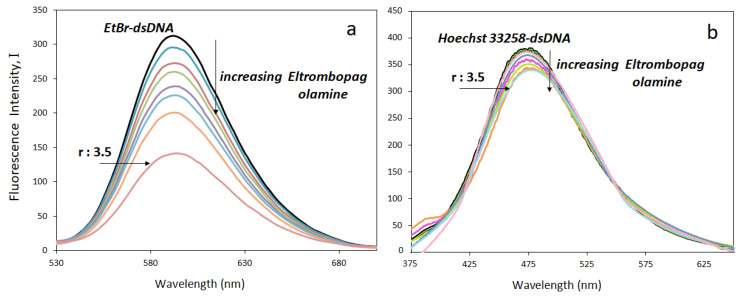
Fluorescence titration of (**a**) EtBr–dsDNA, (**b**) Hoechst-33258–dsDNA mixtures with ELT (r = [ELT]/[dsDNA]).

Fluorescence emission spectra of the ELT-DNA complexes were obtained to calculate the binding constants and also confirm the drug binding mode using the fluorescent dyes ethidium bromide (EtBr) and Hoechst-33258. The former is a strong DNA intercalating agent, whereas the latter is known as a groove binder. Although the fluorescence intensities of EtBr are weak in aqueous media, they increase drastically (almost twenty-five times) upon complexation with DNA due to its intercalation between adjacent DNA base pairs. In replacement experiments, the ELT interacted with solutions containing 50 μM DNA, 5 μM EtBr, and 5 μM Hoechst-33258 at increasing concentrations. EtBr-containing solutions were excited at 294 nm and fluorescence emission spectra were obtained in the range of 530–700 nm. Similarly, Hoechst-33258 solutions were excited at 343 nm and emission spectra were obtained in the range of 375–650 nm. The intercalators replace the EtBr and groove binders replace the Hoechst-33258 in the solutions. As a result, the decrease in the fluorescence emission intensities of either EtBr–DNA or Hoechst–DNA solutions are observed. The lowering of the peak intensities is related to the strength of the drug-DNA interaction. Figure 8a shows the decrease in the fluorescence emission intensities of EtBr-DNA solutions and Figure 8b shows that of Hoechst-33258-DNA solutions.

**Figure 9:**
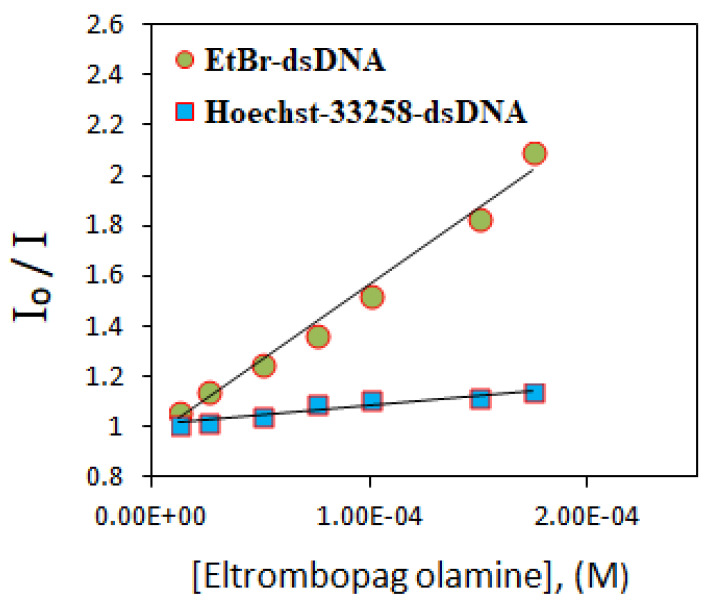
Stern–Volmer plots show the fluorescence quenching of different dye-dsDNA solutions by the increasing addition of ELT. (*R*^2^ = 0.9879 for EtBr–dsDNA, *R*^2^ = 0.9932 for Hoechst-33258–dsDNA).

The fluorescence intensity of the dsDNA-Hoechst-33258 solution almost remained the same upon increasing the ELT concentration. It indicates that Hoechst-33258 replacement did not take place. However, ELT replaces the intercalating agent EtBr. A significant decrease in the emission peak occurs when the concentration of the drug increases. The ELT interacts strongly with DNA by intercalating between the DNA strands. This result was supported by determining the fluorescence quenching capacities according to the Stern–Volmer equation. From the fluorescence titration data, a linear relationship between the emission intensity (*I*_0_/*I*) and ELT concentration was plotted for both EtBr–dsDNA, and Hoechst-33258–dsDNA for comparison (Figure 9).

The *K*_sv_ values were calculated from the slopes of the plots and found to be 6.12 × 10^3^ ± 0.12 M^−1^ and 7.13 × 10^2^ ± 0.20 M^−1^ for EtBr and Hoechst-33258, respectively. A higher *K*_sv_ value of the former shows that EtBr replacement by ELT is 10 times more probable compared with Hoechst-33258. The binding constants (*K*_app_) were estimated using the fluorescence data according to Equation (1):

*K*_EtBr_ . [EtBr] = *K*_app_ . [ELT] (1)

where [ELT] is the concentration of the ELT at half way of fluorescence intensity of EtBr–dsDNA complex. *K*_app_ is defined as the apparent binding constant and found to be 2.86 × 10^5^ M^−1^ ± 0.16 M^−1^ (log *K*_app_ = 5.46 ± 0.09). (*K*_EtBr_ = 1.0 × 10^7^ M^−1^, [EtBr] = 5 μM).

Viscosity measurement is another way of gaining information about the mode of binding. They are sensitive to changes in the length of dsDNA. For the viscosity study, a series of solutions (sample_1- sample_12, Table 3) containing constant concentration of dsDNA and increasing concentrations of ELT were prepared and viscosity data (based on Herschel–Bulkley model parameters) were collected at 25 °C.

**Figure 10:**
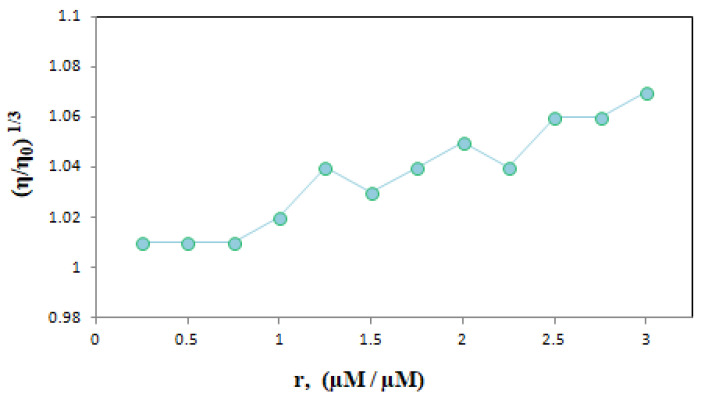
The effect of increasing amount of ELT on the relative viscosity of dsDNA. (r = [ELT]/[dsDNA] = 0.25–3.0).

We graphed the results obtained from the changes in the instantaneous viscosity of the dsDNA solution with increasing ELT amounts based on the data in Table 3 (Figure 10).

Generally, when a small molecule enters between base pairs of DNA, it ruins the double helix structure of DNA and the viscosity of the solution increases [10]. The viscosity remains almost the same when groove binding occurs [22]. Here, we observed that the viscosity of the ELT-DNA mixture increased as a result of an increase in the drug concentration, which is consistent with the intercalation mode of binding.

### 3.3. Molecular Docking & Molecular Dynamics Simulation Studies

Although the experimental studies clearly revealed the binding mode of the drug to be intercalation, in silico studies were also performed to provide more insights into the non-bonding intermolecular interactions, nucleotide regioselectivity, and the role of H-bonding in the binding mechanism of the drug, which is usually concealed in experimental studies. The insight information is very beneficial for drug-developing studies since it enables filtering of ineffective interactions. The strength of ligand-DNA interaction depends on many factors such as chemical structure, molecular weight, shape, functional groups, the number of H-bond donor and acceptor groups of the ligand, and the topology of the binding.

**Figure 11:**
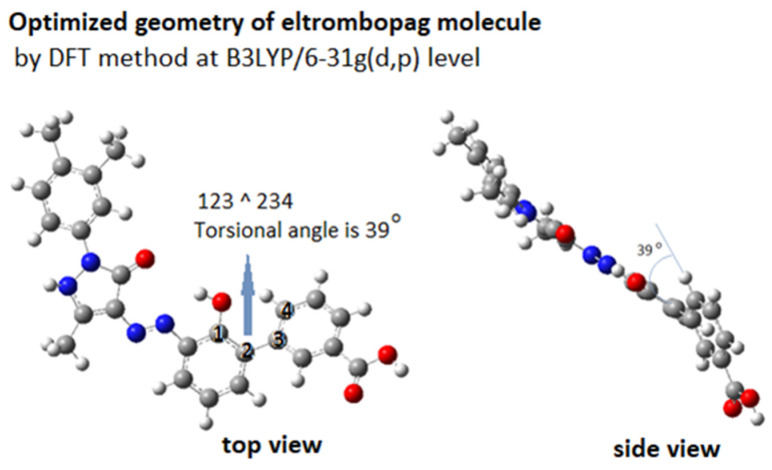
3D structure of ELT molecule.

Figure 11 shows the DFT-optimized geometry of ELT at B3LYP/6-31g(d,p) level. The molecule has an almost planar backbone, with a slightly bent torsional angle of 39° between the benzoic acid moiety and the rest of the molecule. This bent structure makes it more compatible with the helical DNA structure. It has high H-bond forming capacity with 7 H-accepting and 3 H-donating groups.

The stability of ELT at the DNA-bound state was followed by the MD simulations and supported quantitatively by calculating the free energy of binding energies by the molecular docking simulations. In silico studies complemented the multispectroscopic experimental findings, which pointed out the strong binding with a binding constant of approximately 10^5^, consistent with the binding mode of intercalation. The mode of binding is very important since it reveals the effectiveness of systematical silencing/cleavage (by intercalation or groove binding) of the DNA in cancerous cells. Moreover, the attacking of the antineoplastic drug onto the DNA in the cancerous cell and causing apoptosis requires a deeper understanding of the drug’s behavior from a mechanistic point of view at the molecular level.

**Figure 12:**
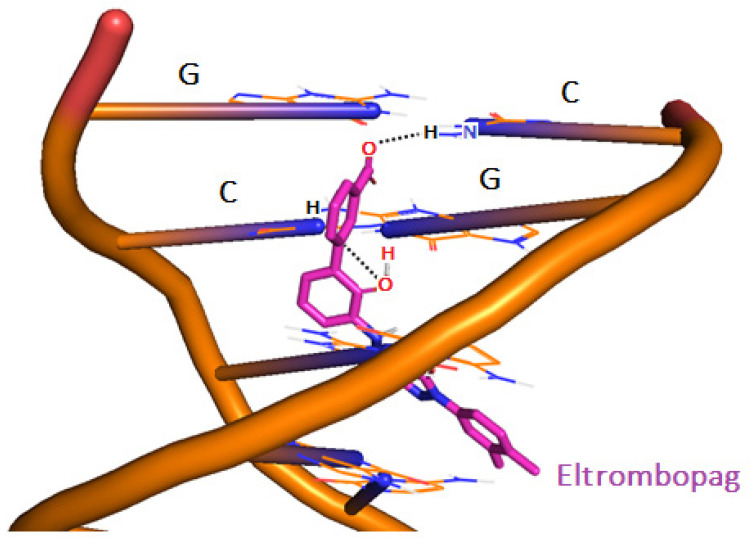
3-D structure of ELT bound to 1BNA via intercalation mechanism at pH 7.4.

Figure 12 illustrates one of the poses of the ELT, taken from the top 80% cluster, where the drug intercalates between the DNA’s helical strands.

Along with the MD results, the cluster analyses of 1000 poses having high docking scores taken from the docking simulations were evaluated. The results showed that the drug forms strong H-bonds at a distance of 1.7 Å, and 80% of the H-bonds were with guanine (G) and cytosine (C) nucleotides of 1BNA and the obtained free energy of binding (docking score) was −15 kcal/mol. The drug unzips and bends the double helical structure of 1BNA. Intercalation mainly occurs via minor grooves of DNA. However, in some minor clusters, the drug also chooses to intercalate via the adjacent phosphodiester sugar backbone of guanine and cytosine, with a relatively lower docking energy of −12.9 kcal/mol (Table 4).

**Figure 13:**
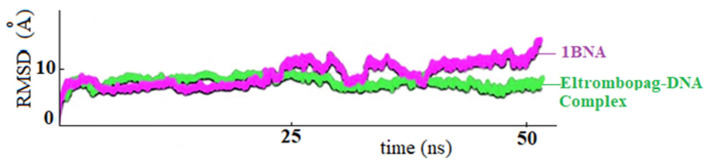
RMSD plots of ELT-BNA complex (in green) and non-ligand bound 1-BNA (in purple), respectively.

In Figure 13, the RMSD plot shows the fluctuation of DNA at drug-bound and unbound states. After the DNA-ligand complex reached equilibrium around 20 nanoseconds, the drug’s coordination reduced the fluctuations, and the system remained stable throughout the simulations between 25–50 ns.

The post-MD hydrogen contact mapping analysis gives detailed information about non-bonded interactions, mainly electrostatic interactions, including H-bond interactions. Short H-bond distances and the strong free energy of binding are usually obtained when the drug intercalates between the DNA. For example, ELT regioselectively binds via its functional groups to guanine and cytosine nucleotides of DNA. Phosphodiester sugar may also contribute to the drug’s intercalation by maneuvering the drug towards guanine and cytosine nucleotides in the DNA’s minor groove.

## 4. Conclusions

In this study, the interaction of ELT with dsDNA was investigated thoroughly by electrochemical, spectroscopic, and molecular docking techniques. The interaction between dsDNA and ELT was performed on a dsDNA biosensor and dsDNA nanobiosensor. The decrease in the oxidation peak currents of dGuo and dAdo upon interaction with ELT at varied concentrations confirmed that the binding occurs at the DNA/rGO/GCE surface.

In voltammetric studies, the peak potentials of dGuo were shifted to the more positive potentials when the interaction time was increased. The increasing concentrations of the dsDNA solutions added to the ELT solution caused a hyperchromic shift of 7 nm in the peaks corresponding to the maximum UV absorption wavelengths. In thermal denaturation studies, the change in the temperature of the dsDNA-ELT complex was about 9.3 °C, close to that of the EtBr-dsDNA complex. When ELT was added to the EtBr-DNA mixture, we observed that ELT replaced the EtBr as reflected in the *K*_sv_ measurements. The increase in the relative viscosity of dsDNA occurred upon addition of ELT into the mixture. All these observations are clear indications that the drug interacts with the DNA strands at the intercalated state. In addition to the experimental findings, molecular docking simulations were carried out to gain more information about the non-bonded interactions. The drug forms strong H-bonds at short distances with the guanine and cytosine nucleotides by intercalating between the DNA strands and remained stable during the course of MD simulations. In some chemotherapeutic research, recognizing specific DNA sequences may be highly important. In such cases, ELT can be used as a positive control since it is a G-C selective drug.

## Figures and Tables

**Table 1:** Physical measurements and instrumentation conditions.

Technical	Property	Instrumentation	ELT Concentration (µM)	dsDNA Concentration (µM)
Absorption spectra	Range of 200–360 nm (with 1 cm quartz cuvettes)	T80 + UV/VIS spectrophotometer	80	20–140 (increasing concentration range)
Absorption spectra (for denaturation profile: temperature from 25 °C to 100 °C)	Range of 200–400 nm (with 1 cm quartz cuvettes)	T80 + UV/VIS spectrophotometer	10 (and EtBr/Hoechst-33258 solution 10 µM)	120
Fluorescence spectra	Range of 530–700 nm (excitation at 294 nm for EtBr studies) Range of 375–650 nm (excitation at 343 nm for Hoechst studies)	Agilent Technologies Spectrofluorometer	12.5–175 (increasing concentration range)	50 (with 5 µM for EtBr and Hoechst-33258)
Viscosity measurements	Rheometer; plate sensor (d = 35 mm, gap = 1 mm)	Haake RheoStress1	30–360 (increasing concentration range)	120

**Table 2:** Thermal denaturation values (°C) of dsDNA in the absence and in the presence of EtBr, Hoechst-33258, and ELT.

Compound	*T*_m_ (°C) ± RSD ^a^	Δ*T*_m_ (°C)
dsDNA	72.2 °C ± 0.07	-
dsDNA + EtBr	83.4 °C ± 0.08	11.6 °C
dsDNA + Hoechst-33258	77.8 °C ± 0.17	5.6 °C
dsDNA + Eltrombopag olamine	81.5 °C ± 0.18	9.3 °C

a: three replicates.

**Table 3:** The Herschel–Bulkey parameters and calculated instantaneous viscosities at 20 s^−1^.

Sample No	Yield Stress (Pa)	K (Pa.s^n^)	n (—)	Instantaneous Viscosity (mPa.s) at 20 s^−1^
Buffer	0.34 ± 0.02	8.06 × 10^−8^ ± 3.01 × 10^−4^	2.77 ± 0.02	16.81
Eltrombopag olamine	0.31 ± 0.04	1.74 × 10^−5^ ± 3.52 × 10^−5^	1.55 ± 0.05	15.60
dsDNA	0.29 ± 0.06	2.34 × 10^−5^ ± 4.78 × 10^−4^	1.78 ± 0.04	14.71
sample 1	0.30 ± 0.06	4.10 × 10^−5^ ± 2.89 × 10^−6^	1.77 ± 0.03	15.41
sample 2	0.30 ± 0.02	4.15 × 10^−5^ ± 4.11 × 10^−6^	1.70 ± 0.06	15.34
sample 3	0.30 ± 0.02	3.26 × 10^−5^ ± 3.48 × 10^−5^	1.76 ± 0.05	15.32
sample 4	0.30 ± 0.05	4.89 × 10^−5^ ± 7.75 × 10^−5^	1.78 ± 0.01	15.51
sample 5	0.30 ± 0.01	3.43 × 10^−4^ ± 6.12 × 10^−3^	1.60 ± 0.01	17.07
sample 6	0.31 ± 0.01	1.17 × 10^−4^ ± 5.65 × 10^−5^	1.50 ± 0.04	16.02
sample 7	0.32 ± 0.01	1.58 × 10^−4^ ± 7.23 × 10^−4^	1.45 ± 0.09	16.61
sample 8	0.33 ± 0.06	1.05 × 10^−4^ ± 6.00 × 10^−3^	1.47 ± 0.03	16.93
sample 9	0.32 ± 0.04	1.01 × 10^−4^ ± 2.42 × 10^−6^	1.46 ± 0.01	16.41
sample 10	0.33 ± 0.08	1.12 × 10^−4^ ± 7.63 × 10^−6^	1.50 ± 0.01	17.00
sample 11	0.33 ± 0.10	1.00 × 10^−4^ ± 2.97 × 10^−5^	1.51 ± 0.06	16.96
sample 12	0.33 ± 0.07	1.35 × 10^−4^ ± 3.91 × 10^−5^	1.51 ± 0.06	17.11

**Table 4:** Eltrombopag post-molecular dynamics hydrogen bond contact analyses.

DNA Location	% of H-Bonds Drug-Nucleotide	Binding Energy (kcal/mol)	H-Bond Distance (Å)
Adenine	-	-	-
Guanine	38.2	−15.4	1.7
Cytosine	41.3	−15.2	1.7
Thymine	-	-	-
Phosphodiester	20.5	−12.9	1.7

## Data Availability

No new data were created or analyzed in this study. Data sharing is not applicable to this article.
